# Genetic Variants Associated With Plasma Lipids Are Associated With the Lipid Response to Niacin

**DOI:** 10.1161/JAHA.117.008461

**Published:** 2018-09-29

**Authors:** Sony Tuteja, Liming Qu, Marijana Vujkovic, Richard L. Dunbar, Jinbo Chen, Stephanie DerOhannessian, Daniel J. Rader

**Affiliations:** ^1^ Department of Medicine Perelman School of Medicine at the University of Pennsylvania Philadelphia PA; ^2^ Department of Biostatistics and Epidemiology Perelman School of Medicine at the University of Pennsylvania Philadelphia PA; ^3^ Department of Genetics Perelman School of Medicine at the University of Pennsylvania Philadelphia PA; ^4^ Cardiometabolic and Lipid Clinic Corporal Michael J. Crescenz VA Medical Center Philadelphia PA; ^5^ ICON plc North Wales PA

**Keywords:** cholesterol, lipids, lipoprotein (a), niacin, pharmacogenetics, statins, Lipids and Cholesterol, Genetic, Association Studies, Pharmacology

## Abstract

**Background:**

Niacin is a broad‐spectrum lipid‐modulating drug, but its mechanism of action is unclear. Genome‐wide association studies have identified multiple loci associated with blood lipid levels and lipoprotein (a). It is unknown whether these loci modulate response to niacin.

**Methods and Results:**

Using data from the AIM‐HIGH (Atherothrombosis Intervention in Metabolic Syndrome with Low HDL/High Triglycerides and Impact on Global Health Outcomes) trial (n=2054 genotyped participants), we determined whether genetic variations at validated loci were associated with a differential change in plasma lipids and lipoprotein (a) 1 year after randomization to either statin+placebo or statin+niacin in a variant‐treatment interaction model. Nominally significant interactions (*P*<0.05) were found for genetic variants in *MVK*,*LIPC, PABPC4, AMPD3* with change in high‐density lipoprotein cholesterol; *SPTLC3* with change in low‐density lipoprotein cholesterol; *TOM1* with change in total cholesterol; *PDXDC1* and *CYP26A1* with change in triglycerides; and none for lipoprotein (a). We also investigated whether these loci were associated with cardiovascular events. The risk of coronary disease related death was higher in the minor allele carriers at the *LIPC* locus in the placebo group (odds ratio 2.08, 95% confidence interval 1.11‐3.90, *P*=0.02) but not observed in the niacin group (odds ratio 0.89, 95% confidence interval 0.48‐1.65, *P*=0.7); *P*‐interaction =0.02. There was a greater risk for acute coronary syndrome (odds ratio 1.85, 95% confidence interval 1.16‐2.77, *P*=0.02) and revascularization events (odds ratio 1.64, 95% confidence interval 1.2‐2.22, *P*=0.002) in major allele carriers at the *CYP26A1* locus in the placebo group not seen in the niacin group.

**Conclusions:**

Genetic variation at loci previously associated with steady‐state lipid levels displays evidence for lipid response to niacin treatment.

**Clinical Trials Registration:**

URL: https://www.clinicaltrials.gov. Unique identifier: NCT00120289.

**Clinical Perspective**

**What Is New?**

- Niacin has been used to modulate the lipid profile, but its mechanism of action is still unclear.
- Using a candidate gene approach, we examined whether genetic loci associated with basal lipid traits were associated with the change in plasma lipid levels in response to niacin.
- We identified common variants in *MVK, LIPC, PABPC4, AMPD3, SPTLC3, PDXDC1,* and *CYP26A1* genes that were suggestive of treatment‐related changes in lipid traits.

**What Are the Clinical Implications?**

- These findings suggest that genetic variation at loci previously associated with steady‐state lipid levels displays evidence for lipid response to niacin treatment.
- After replication of these signals in other larger, independent studies, clinicians may use this information to identify patients who may benefit from niacin therapy.

Niacin has been used in the treatment of dyslipidemias for over 50 years.^1^, ^2^ Niacin reduces total cholesterol (TC), low‐density lipoprotein cholesterol (LDL‐C), triglycerides (TG), and increases high density lipoprotein cholesterol (HDL‐C).^2^ Niacin also lowers lipoprotein (a) [Lp(a)], an independent risk factor for coronary disease.^3^, ^4^ Niacin was one of the first pharmacologic agents shown to reduce the incidence of nonfatal myocardial infarction and cardiac death.^1^ Niacin has also demonstrated beneficial effects on arterial plaque regression in combination with statin therapy.^5^, ^6^ However, in recent clinical outcome trials, the addition of extended‐release niacin to intensive LDL‐C–lowering therapy did not further reduce atherothrombotic events compared with intensive LDL‐C–lowering therapy alone.^7^, ^8^

The mechanisms underlying the lipid‐lowering effects of niacin are still unresolved. Niacin has been shown to increase HDL‐C via reduction of HDL‐apolipoprotein A‐I catabolism and possibly reduction in the expression of cholesteryl ester transfer protein.^9^, ^10^ In vitro studies have suggested that niacin may directly inhibit TG synthesis by inhibition of diacylglycerol acyltransferase in the liver, a key enzyme catalyzing the final step of TG synthesis.^9^ Niacin stimulates the hydroxyl‐carboxylic acid receptor 2 (also known as the niacin receptor) on adipocytes, resulting in a reduction in free fatty acids returning to the liver and decreased assembly of very low‐density lipoproteins.^2^ We previously reported that the coding variant *HCAR2* M317I was not associated with the change in LDL‐C (percentage change −3.7±39.1, −2.6±37.4, −3.5±35.2, *P*=0.58, in the Met‐Met, Met‐Ile, and Ile‐Ile carriers, respectively), HDL‐C (28.2±25.4, 27.6±23.5, 26.1±22.8, *P*=0.62), and TG (−20.9±38.4, −23.0±38.6, −22.5±36.2, *P*=0.50) after 1 year of niacin+statin treatment.^11^ This variant was, however, associated with Lp(a) lowering secondary to niacin (−22.7±35.2, −15.2±40.1, −15.8±37.3, *P*=0.005).^11^

Recent large‐scale genome‐wide association studies (GWAS) have identified 157 loci to be significantly associated with basal fasting lipid traits.^12^ Plasma Lp(a) concentrations are highly genetically regulated with the majority of genetic variation being attributable to the *LPA* locus.^4^, ^13^ It is unknown whether genetic variation at these loci also mediates the effects of lipid‐lowering medications. Individual response to niacin is highly heterogeneous suggesting a potential influence of genetic variation on the pharmacologic response. Although genetic predictors of lipid response to other lipid‐altering drugs (statins,^14^, ^15^ fibrates^16^, ^17^) have been reported, the pharmacogenetics of niacin has not been fully examined.

Here we investigated whether genetic loci associated with basal lipid traits and Lp(a) are associated with the change in plasma lipids and Lp(a) on treatment with ER Niacin in the AIM‐HIGH (Atherothrombosis Intervention in Metabolic Syndrome with Low HDL/High Triglycerides and Impact on Global Health Outcomes) study. We also examined whether these loci were associated with atherothrombotic events in the trial by treatment group.

## Methods

A deidentified data set from the AIM‐HIGH study is available through BioLINCC at the National Heart, Lung and Blood Institute.^18^ Consent language and DNA or patient‐level DNA results will not be made available.

### Ethics Statement

Participants provided written informed consent, and all research was conducted according to the principles outlined in the Declaration of Helsinki. The protocol was approved by the institutional review boards at all participating clinical sites. The genetic substudy was approved by the institutional review board at the University of Pennsylvania.

### AIM‐HIGH Cohort

The AIM‐HIGH study design and baseline characteristics of the participants have been previously published.^7^ Briefly, the trial tested whether extended‐release niacin added to intensive LDL‐lowering therapy including a statin, as compared with intensive, matched LDL‐lowering therapy alone, would reduce the risk of cardiovascular events when LDL was equalized between groups, in an attempt to test whether raising HDL would confer a benefit. The trials enrolled patients with established atherosclerotic cardiovascular disease and atherogenic dyslipidemia (low levels of HDL‐C, elevated TG, and small dense particles of LDL‐C). Of the total 3414 AIM‐HIGH participants, 2054 had complete genetic and phenotype data for the current analysis.

### Genotyping

Participants in the trial were genotyped using the Cardio‐MetaboChip (Illumina, SanDiego, CA). The MetaboChip is a gene‐centric array containing ≈200 000 single nucleotide polymorphisms (SNPs), which were identified through genome‐wide meta‐analyses for metabolic and cardiovascular diseases and phenotypes.^19^ Genotyping was performed on Illumina's iScan System at the Center for Advanced Genomics at the Children's Hospital of Philadelphia. Of the total 3414 AIM‐HIGH participants, 2432 provided DNA for genetic investigation. After initial DNA quality control, 2317 of the 2432 samples were genotyped with a >95% call rate. Cryptic relatedness was estimated by pairwise identity‐by‐descent analysis using PLINK (Shaun Purcell, Harvard, Boston, MA),^20^ resulting in 5 duplicate pairs. The sample of the duplicate pair with lower genotyping call rate was removed. Among 196 725 SNPs on the chip, 19 229 SNPs were monomorphic, and they were removed in subsequent quality control analyses. Multidimensional scaling analysis was used to infer genetic race. Among the 2312 remaining samples, 79 were inferred to have African ancestry, 2101 to have European ancestry, and the rest to represent other races. For the purpose of this study, we performed analyses in participants with European ancestry only.

The lead SNPs primarily associated with each lipid trait were selected from the meta‐analysis published by the Global Lipids Genetics Consortium (Center for Statistical Genetics, Ann Arbor, MI).^12^ If a locus was associated with multiple lipid traits, we only examined the lipid trait primarily associated with that locus. Of the 157 loci validated as blood lipid concentration predictors,^12^ 20 were not found on the Metabochip, and proxies were selected for 18 of them based on linkage disequilibrium in the 1000 Genomes Project pilot data using the Broad institute SNAP tool employing an *r* ^2^ threshold of 0.8 (see Table S1).^21^ We were unable to find proxies for rs964184 in *APOA1* or rs11649653 in *CTF1*. Of the 48 SNPs in the *LPA* and 1 SNP in the *APOE* gene region that are independently associated with Lp(a) concentrations,^13^ 3 SNPS were directly genotyped by MetaboChip, and proxies were found for 5 (Table S1).

Participants were further excluded from the analysis if they were missing lipid data (baseline or year‐1 lipids), yielding 2054 and 1877 for the baseline and 1‐year lipid analyses, respectively. The primary outcome was the percentage change in plasma concentrations of the 4 lipid traits and Lp(a) from baseline to 1 year after treatment with statin+niacin or statin+placebo. We employed a linear model with a SNP‐treatment interaction term to test the additive effect of genotype on the percentage change in lipid traits and Lp(a) at 1 year after the randomization, adjusted for age, sex, body mass index, and treatment arm. We also examined the baseline prerandomization plasma concentrations of HDL‐C, LDL‐C, TC, TG, and Lp(a). Log transformations were carried out on nonnormally distributed variables. Logistic regression models were fit to evaluate the effect of genotype on coronary artery dsease (CAD) outcomes in an interaction model. For the single‐marker lipid analysis, *P* values were adjusted for multiple testing using the Bonferroni approach based on 320 hypotheses (58, 30, 37, 27, 8 SNPs for HDL‐C, LDL‐C, TC, TG, and Lp(a), respectively, interrogated at 2 time points), yielding a statistical significance threshold of 0.0002. Because no SNPs achieved this *P* value in the interaction analysis, top hits with a *P* value of <0.05 were reported because these SNPs have previously been associated with lipid traits at genome‐wide levels of significance, and our analyses represent further characterization of each of these established loci.

A poststudy power analysis was performed for the association of *LIPC* SNP rs1532085 and change in HDL‐C using a bootstrap method, resampling with replacement 10 000 times using the linear regression function in R v3.4.4.^22^ At α thresholds of *P*=0.05 and 0.0002, the study had a power of 87% and 26%, respectively, to detect this association.

## Results

### Study Population and Lipoprotein Changes

The clinical and demographic characteristics for the AIM‐HIGH population that provided DNA during the course of the study as compared with the whole cohort are provided in Table 1. In the whole cohort as previously published,^7^ treatment with niacin resulted in a significant increase in HDL‐C and a significant decrease in triglyceride concentrations as compared with the placebo group. The change in lipids we observed in the genetic subgroup was similar to that in the whole AIM‐HIGH cohort. In a separate analysis of AIM‐HIGH, the addition of niacin resulted in a significant reduction in Lp(a) levels by 21% in the statin+ER niacin group compared with 5.9% in the statin+placebo group (*P*<0.05).^23^

**Table 1:** Clinical and Demographic Characteristics of the Study Participants

Mean±SD, n (%)	Genetic Subgroup		Total AIM‐HIGH Study	
	Statin+Placebo (n=1020)	Statin+ER Niacin (n=1034)	Statin+Placebo (n=1696)	Statin+ER Niacin (n=1718)
Age, y	64.0±8.7	64.6±8.7	63.7±8.7	63.7±8.8
Sex, female	164 (16.1%)	158 (15.3%)	251 (14.8%)	253 (14.7%)
Body mass index, kg/m^2^	31.2±5.3	31.6±5.7	30.9±5.2	31.5±5.5^a^
History of myocardial infarction	556 (54.5)	554 (53.6)	955 (56.3)	968 (56.3)
History of stroke	240 (23.5)	244 (23.6)	362 (21.3)	358 (20.8)
History of hypertension	741 (72.6)	787 (76.1)	1189 (70.1)	1250 (72.8)
History of diabetes mellitus	341 (33.4)	351 (34.0)	570 (33.6)	588 (34.2)
Baseline HDL‐C, mg/dL	35.1±5.6	34.6±5.6^a^	35.3±5.9	34.8±5.9^a^
Baseline LDL‐C, mg/dL	74.6±22.2	73.5±22.0	75.8±24.3	76.2±25.7
Baseline TC, mg/dL	146.0±26.8	144.8±26.9	145.2±26.6	145.4±28.2
Baseline TG, (mg/dL), median (IQR)	162 (133‐215)	166 (131‐217)	162 (128‐218)	164 (127‐218)
Baseline lipoprotein (a) (nmol/L), median (IQR)	32 (13‐118)	36 (14‐132)	32.7 (13.1‐122.6)	36.1 (13.5‐126.6)
Change in LDL‐C	−4.5 (−20.5, 13.9)	−9.5 (−28.0, 12.3)^a^	−4.25 (−20.57, 15.70)	−10.00 (−28.00, 12.68)^b^
Change in HDL‐C	9.4 (0, 18.8)	25.0 (11.4 to 39.5)^b^	9.09 (0.00, 18.92)	23.33 (10.34, 39.29)^b^
Change in TC	0 (−12.0, 11.1)	−5.0 (−16.4, 8.4)^b^	−0.55 (−11.81, 11.59)	−5.19 (−16.17, 8.00)^b^
Change in TG	−4.4 (−24.6, 20.9)	−29.3 (−48.0, −6.4)^b^	−5.03 (−25.61, 20.77)	−28.24 (−46.61, −3.13)^b^
Change in Lp(a)	−7.5 (−25.9, 11.3)	−19.7 (−38.5, −0.6)	−7.0 (−25, 13.0)	−20.0 (−39.0, 1.0)

Change in lipid traits reported as median percentage change from baseline to 1 year (IQR). AIM‐HIGH indicates Atherothrombosis Intervention in Metabolic Syndrome with Low HDL/High Triglycerides and Impact on Global Health Outcomes; ER, extended‐release; HDL‐C, high‐density lipoprotein cholesterol; IQR, interquartile range; LDL‐C, low‐density lipoprotein cholesterol; Lp(a), lipoprotein (a); TC, total cholesterol; TG, triglycerides.

a *P*<0.05 compared with placebo group.

b *P*<0.0001.

### Baseline Associations

The SNP genotypes, their chromosomal locations, and the nearest genes and their allele frequencies in the AIM‐HIGH trial are shown in Table S1. Allele frequencies were comparable to those previously reported by the Global Lipids Genetics Consortium^12^ and in a recent GWAS for Lp(a).^13^ We tested associations of these SNPs with baseline HDL‐C, LDL‐C, TC, TG, and Lp(a). All (*P*<0.05) associations are shown in Table 2. The threshold for Bonferroni correction for multiple comparisons was an adjusted α level of 0.0002. At this level we replicated associations with HDL‐C at 1 locus and LDL‐C at 1 locus. We also replicated the association of 8 variants within the *LPA* locus with Lp(a) levels. Given the size of our genotyped cohort, this is consistent with expectations based on power. Furthermore, more than 90% of the AIM‐HIGH cohort was taking a statin at baseline and had a median LDL‐C of 74 mg/dL, which may have obscured additional baseline associations.

**Table 2:** Significant Genotypic Associations With Lipid Traits at Baseline

SNP	Trait	Locus	Chr	MAF	N	β	SE	*P* Value
rs3764261	HDL‐C	*CETP*	16	0.30	2054	0.178	0.032	2.4×10^–8^
rs1532085	HDL‐C	*LIPC*	15	0.37	2054	0.106	0.023	0.0004
rs3136441	HDL‐C	*LRP4*	11	0.12	2054	0.130	0.045	0.0037
rs581080	HDL‐C	*TTC39B*	9	0.19	2054	−0.087	0.037	0.018
rs838880	HDL‐C	*SCARB1*	12	0.31	2054	0.063	0.031	0.042
rs7239867	HDL‐C	*LIPG*	18	0.17	2054	−0.077	0.038	0.046
rs6450176	HDL‐C	*ARL15*	5	0.26	2054	−0.064	0.032	0.048
rs629301	LDL‐C	*SORT1*	1	0.20	2054	−0.153	0.039	8.05×10^–5^
rs4299376	LDL‐C	*ABCG 5/8*	2	0.31	2054	0.082	0.034	0.015
rs10490626	LDL‐C	*INSIG2*	2	0.072	2054	−0.141	0.060	0.018
rs364585	LDL‐C	*SPTLC3*	20	0.39	2054	−0.063	0.032	0.046
rs4253772	TC	*PPARA*	22	0.11	2054	0.138	0.050	0.0059
rs1169288	TC	*HNF1A*	12	0.32	2054	0.090	0.033	0.0072
rs11065987	TC	*BRAP*	12	0.44	2054	−0.082	0.031	0.0090
rs2642442	TC	*MOSC1*	1	0.31	2054	−0.071	0.033	0.032
rs2954029	TG	*TRIB1*	8	0.44	2054	−0.113	0.031	0.0003
rs1260326	TG	*GCKR*	2	0.43	2054	0.112	0.031	0.0003
rs2131925	TG	*ANGPTL3*	1	0.33	2054	−0.089	0.033	0.0074
rs12678919	TG	*LPL*	8	0.073	2054	−0.131	0.060	0.028
rs174546	TG	*FADS 1‐2‐3*	11	0.34	2054	0.072	0.033	0.029
rs7769879	Lp(a)	*SLC22A3*	6	0.39	2054	0.351	0.032	7.03×10^–28^
rs539298	Lp(a)	*SLC22A3*	6	0.47	2054	−0.273	0.031	1.99×10^–18^
rs4252109	Lp(a)	*PLG*	6	0.29	2054	−0.290	0.034	1.32×10^–17^
rs2504927	Lp(a)	*SLC22A3*	6	0.43	2054	−0.250	0.032	4.59×10^–15^
rs394352	Lp(a)	*SLC22A3*	6	0.29	2054	−0.245	0.034	7.30×10^–13^
rs3798221	Lp(a)	*LPA*	6	0.19	2054	−0.282	0.039	1.00×10^–12^
rs986666	Lp(a)	*SLC22A3*	6	0.20	2054	−0.155	0.039	8.10×10^–5^
rs2457561	Lp(a)	*SLC22A3*	6	0.19	2054	−0.154	0.041	0.00015

Chr indicates chromosome; HDL‐C, high‐density lipoprotein cholesterol; LDL‐C, low‐density lipoprotein cholesterol; Lp(a), lipoprotein (a); MAF, minor allele frequency; SE, standard error; SNP, single nucleotide polymorphism; TC, total cholesterol; TG, triglycerides.

### Association With Lipid Traits at 1 Year

We tested each SNP for interaction with niacin in modulating the change in lipid traits at 1 year. None of the interaction results reached our adjusted *P* value corrected for multiple testing. Nominally significant SNP‐treatment interactions were found for *MVK*,* LIPC, PABPC4,* and *AMPD3* with change in HDL‐C; *SPTLC3* with change in LDL‐C; *TOM1* with change in TC; *PDXDC1* and *CYP26A1* with change in TG at 1 year (Table 3). No significant SNP‐treatment interactions were found for Lp(a).

**Table 3:** Nominally Significant Gene‐Treatment Interactions in Association With the Change in Lipid Traits From Baseline to 1 Year

SNP	Trait	Locus	Chr	MAF	N	β	SE	*P* Value_Interaction
rs10850443	HDL‐C	*MVK*	12	0.47	1877	−0.170	0.059	0.0039
rs1532085	HDL‐C	*LIPC*	15	0.36	1877	0.176	0.061	0.0040
rs4660293	HDL‐C	*PABPC4*	1	0.23	1877	0.178	0.071	0.013
rs2923084	HDL‐C	*AMPD3*	11	0.18	1877	−0.156	0.077	0.043
rs364585	LDL‐C	*SPTLC3*	20	0.39	1877	−0.139	0.067	0.039
rs138777	TC	*TOM1*	22	0.34	1877	0.133	0.066	0.044
rs3198697	TG	*PDXDC1*	16	0.39	1877	0.175	0.062	0.0047
rs2068888	TG	*CYP26A1*	10	0.440	1877	−0.120	0.061	0.049

Chr indicates chromosome; HDL‐C, high‐density lipoprotein cholesterol; LDL‐C, low‐density lipoprotein cholesterol; MAF, minor allele frequency; SE, standard error; SNP, single nucleotide polymorphism; TC, total cholesterol; TG, triglycerides.

Lipid levels by genotype are reported in Table 4. For the *MVK* gene, there was no change by genotype in HDL‐C levels in the placebo group, but there was a nominally significant genotype effect in the niacin group. At the *LIPC* locus, a change in HDL‐C by genotype was observed in the placebo group, but this effect was diminished in the niacin group. In the AIM‐HIGH trial the placebo group achieved an overall 9% increase in HDL‐C at the end of the first year, possibly because they received a small dose of immediate‐release niacin to mask the treatment assignment, a dose previously shown to significantly raise HDL‐C.^24^ At the *PABPC4* locus a change in HDL‐C by genotype was observed in the placebo group that was not observed in the niacin group. The change in HDL‐C was nominally significant in the interaction model for *AMPD3* but no longer significant within each treatment strata.

**Table 4:** Levels of Quantitative Traits at 1 Year by Genotype and Treatment Arm at Loci With a Nominally Significant Interaction

Gene/Chr/SNP	Alleles				Placebo+Statin						Niacin+Statin					
					Baseline		Year 1		% Change	Absolute Change	Baseline		Year 1		% Change	Absolute Change
	MAF	Minor/Major	Trait	GT	N	Mean (SD)^a^	N	Mean (SD)	Mean (SD)	Mean (SD)	N	Mean (SD)	N	Mean (SD)	Mean (SD)	Mean (SD)
*MVK*	0.47	C/T	HDL	TT	280	34.9 (5.4)	256	38.1 (7.9)	9.1 (15.9)	3.1 (5.7)	304	34.8 (5.7)	277	45.4 (12.1)	30.8 (25.3)	10.7 (9.3)
12				TC	474	35.4 (5.5)	435	39.1 (7.4)	10.6 (15.3)	3.6 (5.3)	517	34.5 (5.4)	471	43.5 (10.6)	26.4 (23.1)	9.1 (8.1)
rs10850443				CC	265	34.7 (5.4)	245	38.2 (7.2)	10.5 (16.0)	3.5 (5.3)	211	34.5 (5.8)	189	43.4 (11.2)	26.4 (24.1)	9.0 (8.8)
		*P* Value by GT							0.22	0.26					0.012	0.007
*LIPC*	0.36	A/G	HDL	GG	404	34.6 (5.7)	376	38.2 (7.5)	11.7 (15.9)	3.9 (5.3)	429	34.5 (5.8)	387	43.2 (11.5)	25.8 (23.3)	8.9 (8.6)
15				GA	465	35.1 (5.3)	428	38.4 (7.1)	9.4 (15.3)	3.2 (5.3)	472	34.5 (5.5)	430	44.5 (11.2)	29.0 (24.7)	10.0 (8.6)
rs1532085				AA	151	36.1 (5.3)	133	39.6 (8.2)	8.1 (15.4)	3.0 (6.0)	132	35.1 (5.3)	121	44.9 (9.7)	29.2 (23.6)	10.0 (8.2)
		*P* Value by GT							0.006	0.026					0.037	0.031
*PABPC4*	0.23	G/A	HDL	AA	617	35.1 (5.4)	567	38.8 (7.3)	11.0 (5.6)	3.7 (5.4)	580	34.9 (5.8)	530	44.4 (11.2)	27.2 (23.5)	9.4 (8.6)
1				AG	361	35.3 (5.5)	332	38.3 (7.7)	9.1 (15.6)	3.1 (5.5)	390	34.3 (5.4)	354	43.8 (11.3)	28.4 (24.6)	9.7 (8.8)
rs4660293				GG	42	32.9 (5.8)	38	34.7 (6.3)	7.3 (16.4)	2.1 (5.6)	64	34.0 (5.5)	55	43.8 (10.2)	28.7 (25.0)	9.7 (8.5)
		*P* Value by GT							0.029	0.031					0.33	0.41
*AMPD3*	0.18	G/A	HDL	AA	664	35.1 (5.4)	617	38.4 (7.6)	9.8 (15.9)	3.3 (5.5)	698	34.6 (5.7)	633	44.2 (11.3)	28.2 (23.5)	9.7 (8.6)
11				AG	323	35.1 (5.7)	292	38.8 (7.3)	10.9 (15.2)	3.7 (5.3)	300	34.6 (5.5)	275	43.9 (11.1)	27.7 (25.5)	9.5 (8.7)
rs2923084				GG	33	33.4 (5.1)	28	36.8 (6.7)	9.9 (15.9)	3.2 (5.0)	36	34.9 (5.3)	31	41.2 (9.2)	18.2 (18.5)	6.3 (6.9)
		*P* Value by GT							0.28	0.30					0.26	0.2
*SPTLC3*	0.39	A/G	LDL	GG	376	76.1 (22.8)	342	71.3 (19.1)	−2.7 (28.6)	−5.6 (24.1)	406	74.2 (21.8)	361	68.9 (20.7)	−1.1 (37.4)	−5.4 (25.5)
20				GA	482	72.3 (21.0)	448	70.6 (18.5)	1.2 (33.1)	−3.3 (23.9)	479	73.1 (22.5)	443	65.6 (19.8)	−4.5 (38.1)	−8.5 (26.3)
rs364585				AA	162	72.4 (22.4)	144	69.2 (7.3)	1.2 (34.0)	−3.6 (24.5)	149	73.2 (22.5)	134	65.1 (15.1)	−5.2 (37.3)	−9.0 (23.8)
		*P* Value by GT							0.08	0.22					0.11	0.043
*TOM1*	0.34	A/G	TC	GG	456	144.4 (24.6)	413	143.9 (24.6)	1.5 (19.6)	−0.7 (29.5)	462	146.2 (27.6)	422	138.8 (27.5)	−3.5 (21.2)	−8.3 (32.0)
22				GA	441	145.4 (26.4)	408	143.7 (24.4)	0.8 (19.3)	−1.9 (28.5)	437	142 (25.7)	392	136.5 (26.2)	−1.5 (22.2)	−5.2 (31.4)
rs138777				AA	123	149.9 (30.2)	117	144.2 (8.4)	−0.08 (25.7)	−5.5 (38.1)	135	147.3 (36.2)	128	141 (26.4)	−2.2 (21.3)	−6.5 (33.9)
		*P* Value by GT							0.35	0.38					0.14	0.12
*PDXDC1*	0.39	T/C	TG	CC	392	156 (128, 212)	365	156 (119, 207)	−4 (−23, 21)	−7 (−36, 32)	359	178 (140, 235)	331	117 (85, 160)	−32.0 (−49, −13)	−54 (−97, −21)
16				CT	471	165 (134, 216)	431	158 (123, 206)	−3 (−26, 24)	−5 (−43, 35)	516	162 (130, 213)	466	120 (83, 171)	−29.4 (−48, −1.5)	−46 (−80, −2)
rs3198697				TT	157	173 (136, 222)	142	154 (113, 207)	−9 (−29, 11)	−16 (−47, 20)	159	156 (127, 203)	145	124 (85, 167)	−24.5 (−46, −3)	−38 (−77, −5)
		*P* Value by GT							0.13	0.077					0.034	0.015
*CYP26A1*	0.44	A/G	TG	GG	331	165 (135, 224)	306	161 (121, 213)	−4 (27, 20)	−7 (−44, 30)	311	165 (129, 225)	288	131 (90, 187)	−23 (−42, 2)	−36 (−76, 3)
10				GA	493	159 (131, 203)	451	155 (118, 206)	−4 (−23, 22)	−7 (−44, 33)	525	171 (135, 217)	473	114 (82, 161)	−32 (−51, −11)	−54 (−92, −18)
rs2068888				AA	196	161 (133, 222)	181	157 (123, 204)	−7 (−27, 21)	−11 (−45, 32)	198	161 (126, 210)	181	115 (83, 157)	−32 (−48, −10)	−48 (−85, −16)
		*P* Value by GT							0.40	0.59					<0.0001	0.001

Chr indicates chromosome; GT, genotype; HDL, high‐density lipoprotein cholesterol; IQR, interquartile range; LDL, low‐density lipoprotein cholesterol; MAF, minor allele frequency; SNP, single nucleotide polymorphism; TC, total cholesterol; TG, triglycerides.

a TG reported as median (IQR).

The change in LDL‐C was greater in minor allele carriers at the *SPTLC3* locus in the niacin group but not in the placebo group. Major allele carriers at the *PDXDC1* locus had a greater decrease in TG if they were treated with niacin but not with placebo. Last, minor allele carriers at the *CYP26A1* locus had the largest decrease in TG levels in the niacin group but not in the placebo group. The minor allele for *CYP26A1* was associated with lower TG at baseline but a larger decrease in TG at 1 year in the niacin group only (*P*<0.0001) (Table 4). The placebo group saw a nonsignificant change in TG from baseline regardless of the *CYP26A1* genotype.

### Cardiovascular Outcomes

We also tested whether the SNPs nominally significant in the interaction model for lipid traits were associated with the risk of developing atherosclerotic events during the 3‐year follow‐up in the AIM‐HIGH trial. The interactions of treatment and SNP on the primary cardiovascular end point (defined in the AIM‐HIGH trial as the composite of death from CAD, nonfatal myocardial infarction, ischemic stroke, hospitalization for acute coronary syndrome, and symptom‐driven revascularization) and the individual components of the primary end point are reported in Table 5.

**Table 5:** Nominally Significant Lipid Traits Associated With Coronary Artery Disease Events

Gene	*MVK*	*LIPC*	*PABPC4*	*AMPD3*	*SPTLC3*	*TOM1*	*PDXDC1*	*CYP26A1*
SNP	rs10850443	rs1532085	rs4660293	rs2923084	rs364585	rs138777	rs3198697	rs2068888
Lipid trait	HDL‐C	HDL‐C	HDL‐C	HDL‐C	LDL‐C	TC	TG	TG
N	2054	2054	2054	2054	2054	2054	2054	2054
Composite end point								
OR	1.06	1.17	0.88	0.93	0.94	1.23	1.05	0.82
SE	0.12	0.12	0.15	0.16	0.12	0.12	0.12	0.12
*P* Value×int	0.63	0.20	0.41	0.64	0.62	0.10	0.67	0.11
Death from CHD								
OR	1.29	2.09	0.88	2.17	0.48	1.51	1.09	1.63
SE	0.32	0.32	0.41	0.35	0.37	0.32	0.32	0.33
*P* Value×int	0.43	0.021^a^	0.76	0.028^a^	0.05	0.20	0.78	0.13
Overall death								
OR	0.87	1.14	1.20	1.32	0.75	1.11	0.82	1.15
SE	0.21	0.21	0.25	0.25	0.22	0.22	0.22	0.21
*P* Value×int	0.51	0.53	0.46	0.27	0.19	0.63	0.35	0.50
Myocardial infarction								
OR	1.06	1.17	0.87	0.93	0.99	1.26	0.97	0.87
SE	0.19	0.19	0.24	0.25	0.20	0.19	0.20	0.20
*P* Value×int	0.74	0.42	0.58	0.76	0.94	0.23	0.88	0.49
Ischemic events								
OR	1.07	0.80	1.36	0.62	1.80	0.77	0.50	0.93
SE	0.42	0.45	0.50	0.64	0.43	0.48	0.50	0.44
*P* Value×int	0.87	0.62	0.55	0.45	0.17	0.59	0.17	0.86
Hospitalization from ACS								
OR	1.14	0.92	1.13	0.88	0.80	0.93	1.13	0.56
SE	0.22	0.23	0.27	0.29	0.23	0.24	0.22	0.24
*P* Value×int	0.55	0.73	0.65	0.67	0.34	0.76	0.60	0.017^a^
Symptom driven revascularization								
OR	1.22	1.11	0.79	0.71	1.04	1.27	1.10	0.60
SE	0.15	0.15	0.19	0.21	0.15	0.15	0.15	0.16
*P* Value×int	0.18	0.50	0.23	0.10	0.80	0.11	0.54	0.0013^a^

ACS indicates acute coronary syndrome; CHD, congenital heart disease; HDL, high‐density lipoprotein cholesterol; LDL, low‐density lipoprotein cholesterol; OR, odds ratio; *P*‐value×int, interaction *P*‐value; SE, standard error; SNP, single nucleotide polymorphism; TC, total cholesterol; TG, triglycerides.

a *P* < 0.05.

A SNP‐treatment interaction was found for *LIPC* and cardiovascular death. Homozygous minor allele carriers in the placebo group experienced the highest rate of CAD death (4%) versus heterozygous carriers (2.3%) and homozygous major allele carriers (1.0%) (odds ratio [OR]=2.08; 95% confidence interval 1.11‐3.90; *P*=0.02) (Table 6). There was no difference in the rate of CAD death by *LIPC* genotype among the subjects receiving niacin (OR=0.89, 95% confidence interval 0.48‐1.65, *P*=0.71). There was also a SNP‐treatment interaction for *AMPD3* and CAD death. Minor allele carriers of the *AMPD3* had the lowest HDL‐C levels at baseline and saw the smallest change in HDL‐C levels at 1 year in both the niacin and placebo groups. However, the subjects homozygous for the minor allele in the placebo group had the highest rate of CAD death (9.1%) versus heterozygous carriers (2.5%) and homozygous major allele carriers (1.4%) (OR=2.16, 95% confidence interval 1.09‐4.2, *P*=0.03). There was no difference in CAD death by *AMPD3* genotype in the niacin group (OR=1.8, 95% 0.96‐3.45, *P*=0.07).

**Table 6:** Coronary Events by Genotype and Treatment Arm at Loci With a Nominally Significant Interaction

Gene/Chr/SNP	Alleles			Placebo+Statin				Niacin+Statin		
	MAF	Minor/Major	Trait	GT	N	Frequency of Events	Odds Ratio (95% CI)	N	Freqency of Events	Odds Ratio (95% CI)
*LIPC*	0.36	A^a^/G	CAD Death	GG	404	4 (1%)	2.08 (1.11, 3.90)	429	9 (2.1%)	0.89 (0.48, 1.65)
15				AG	465	10 (2.2%)		472	13 (2.8%)	
rs1532085				AA	151	6 (4.0%)		132	2 (1.5%)	
			*P* Value by GT				0.02			0.71
*AMPD3*	0.18	G^a^/A	CAD Death	AA	664	9 (1.4%)	2.16 (1.09, 4.29)	698	11 (1.6%)	1.82 (0.96, 3.45)
11				AG	323	8 (2.5%)		300	12 (4.0%)	
rs2923084				GG	33	3 (9.1%)		36	1 (2.8%)	
			*P* Value by GT				0.03			0.07
*CYP26A1*	0.44	A/G^a^	ACS	GG	331	21 (6.3%)	1.85 (1.16, 2.77)	311	15 (4.8%)	1.20 (0.79, 1.85)
10				GA	493	17 (3.5%)		525	25 (4.8%)	
rs2068888				AA	196	4 (2.0%)		198	6 (3.0%)	
			*P* Value by GT				0.02			0.39
*CYP26A1*	0.44	A/G^a^	Revascularization	GG	331	41 (12.4%)	1.64 (1.20, 2.22)	311	29 (9.3%)	0.98 (0.71, 1.32)
10				GA	493	50 (10.1%)		525	49 (9.3%)	
rs2068888				AA	196	7 (3.6%)		198	19 (9.6%)	
			*P* value by GT				0.002			0.83

*P*‐value within each treatment group determined by logistic regression adjusted for age, sex, BMI. ACS indicates acute coronary syndrome; BMI, body mass index; CAD, coronary artery disease; Chr, chromosome; CI, confidence interval; GT, genotype; MAF, minor allele frequency; SNP, single nucleotide polymorphism;.

a Risk allele.

A nominally significant SNP‐treatment interaction was found for *CYP26A1* and acute coronary syndrome and symptom‐driven revascularization. As mentioned above, the minor allele for *CYP26A1* was associated with lower TG at baseline but a larger decrease in TG at 1 year in the niacin group only (Table 4), whereas the placebo group saw no change in TG from baseline regardless of *CYP26A1* genotype. Major allele carriers had a higher rate of cardiac events—both acute coronary syndrome and symptom‐driven revascularization—in the placebo group only (Table 6). There were no differences in cardiac events by *CYP26A1* genotype in the niacin group.

## Discussion

In this study we evaluated whether genetic loci associated with plasma lipid levels and Lp(a) could also be pharmacogenetic markers of lipid response to niacin. We present data that common variants in *MVK, LIPC, PABPC4, AMPD3, SPTLC3, PDXDC1,* and *CYP26A1* genes were associated with treatment‐related changes in lipid traits as observed 1 year following randomization in the AIM‐HIGH trial. Additionally, *LIPC, AMPD3,* and *CYP26A1* variants were associated with cardiovascular events in the placebo‐treated patients but not in the group receiving niacin.

In the AIM‐HIGH trial, subjects in the placebo group received a small dose of immediate‐release niacin to mask the treatment assignment; accordingly, the placebo group achieved an overall 9% increase in HDL‐C at the end of the first year compared with 23% in the statin+niacin group. At the *LIPC* locus, rs1532085 was significantly associated with the change in HDL‐C in the group receiving statin+placebo treatment, but treatment with niacin appeared to overcome the genotype effect. Most intriguing is that homozygous minor allele carriers in the statin+placebo group who saw the smallest change in HDL‐C had the highest frequency of cardiovascular‐related death (OR=2.08, *P*=0.02), which was not observed in the statin+niacin group (OR=0.89, *P*=0.7; *P*‐interaction=0.02).

Hepatic lipase, encoded by *LIPC*, is a plasma lipolytic enzyme that hydrolyzes triglycerides and phospholipids in chylomicron remnants, intermediate‐density lipoproteins, and HDL.^25^ Hepatic lipase is an important determinant of plasma HDL‐C, converting the large, buoyant, phospholipid‐rich HDL_2_ to small, dense HDL_3._ 26 The presence of the C‐allele in a common promoter polymorphism in *LIPC* (−514 C>T) is associated with greater hepatic lipase activity, small, dense LDL‐C particles, and lower levels of the atheroprotective HDL_2_ levels.^27^, ^28^ Lipid‐lowering therapies, including niacin, have been shown to decrease hepatic lipase activity, increase HDL_2_ and coronary disease regression, with a significantly greater effect in the CC (carrying two copies of the C allele at −514 C>T) subjects.^28^ Although the rs1532085 is not in linkage disequilibrium with the −514 C>T variant, there is previous evidence for differential lipid‐lowering responses by genotype at the *LIPC* locus.^28^ In the genome‐wide analysis published by the Global Lipids Consortium, the minor allele of rs1532085 was associated with higher HDL‐C plasma concentrations and decreased transcript expression in liver tissue.^29^ In the current study the minor allele of rs1532085 was also associated with higher HDL‐C concentration at baseline, but at 1 year, the change in HDL‐C was smaller in the minor allele carriers randomized to the statin+placebo arm. It may seem paradoxical that a variant associated with higher HDL‐C may be associated with an increased risk for CAD. However, it has been previously demonstrated that a loss‐of‐function variant in *SCARB1* (P376L), coding for the scavenger receptor B1, the major receptor for HDL‐C, was associated with significantly increased plasma HDL‐C and an increased risk of CAD.^30^ The growing consensus surrounding HDL biology indicates that HDL function and cholesterol flux may be more important than steady‐state concentrations of HDL‐C.^31^ Although rs1532085 has not previously been associated with CAD, 2 other variants in *LIPC*, rs588136 (*P*=3.7×10^−4^) and rs1800588 (*P*=4.7×10^−4^) have been nominally associated with CAD.^32^, ^33^

Previous GWAS studies have shown that the minor allele carriers of *CYP26A1,* rs2068888, have significantly lower TG levels.^29^ In our study baseline TG was not different at this SNP because of the sample size of our study, but the change in TG levels was different by genotype in the niacin‐treated subjects but not in the subjects receiving statin alone. This SNP has also been nominally associated with CAD (*P*=5.4×10^−5^)^32^, ^34^ and atrial fibrillation (*P*=0.0064).^34^, ^35^ Cytochrome P450 26A1 is an endoplasmic reticulum protein with high expression in the liver that metabolizes all‐*trans*‐retinoic acid, thereby regulating cellular levels of retinoic acid.^36^ Retinoic acid binds the retinoid x receptor,^37^ which plays an important role in lipid metabolism by itself and also by heterodimerizing with other well‐known nuclear receptors such as peroxisome proliferator‐activated receptors, farnesoid x receptor, and liver x receptor.^38^, ^39^ Retinoic acid treatment has been in shown to reduce TG levels in mice, whereas retinoid x receptor deletion induced the synthesis of TG.^39^ In patients receiving oral retinoids for the treatment of dermatological conditions, 44% experienced elevations in plasma triglycerides.^40^ Treatment with bexarotene, a third‐generation retinoid used in the treatment of T‐cell lymphoma, results in hyperlipidemia in most patients, and fatal cases of cholestasis and pancreatitis have been reported.^41^ Recent GWAS studies investigating the polygenic genetic signal for the basis of CAD and several cardiovascular disease risk factors have shown that the liver x receptor/retinoid x receptor and farnesoid x receptor/retinoid x receptor activation pathways are the top pathways enriched by CAD SNPs.^42^

We were able to replicate the association with known variants at the *LPA* locus with baseline Lp(a) concentrations in AIM‐HIGH. It was disappointing that none of these variants was associated with the change in Lp(a) in response to niacin. Thus, the mechanism by which niacin lowers Lp(a) is still unclear.

Previous GWAS studies have identified loci associated with statin response.^14^, ^15^ A meta‐analysis has identified 4 genetic loci, *APOE* (rs445925), *LPA* (rs10455872), *SORT1* (rs646776), and *SLCO1B1* (rs2900478), associated with percentage LDL‐C reduction following statin therapy at a genome‐wide level.^14^ Only *CETP* was identified with the change in HDL‐C in response to statins.^15^ We did not find *APOE, LPA, SORT1,* or *CETP* to be associated with niacin response in our study. A candidate gene study found a SNP at the *APOA1* (rs964184) locus associated with fenofibrate response.^16^ This SNP was not genotyped in our cohort, so we were unable to determine whether it also mediated response to niacin. A pharmacogenetic analysis using a genome‐wide approach in the ACCORD (Action to Control Cardiovascular Risk in Diabetes) trial identified *HSD17B3, SMAD3,* and *IPO11* as genetic markers of fenofibrate response.^17^

There are several limitations to this study. First, the sample size in our study is small. To detect the association of *LIPC* SNP rs1532085 and change in HDL‐C, the study was powered at 87% and 26% for α thresholds of 0.05 and 0.0002, respectively. The latter threshold using the Bonferroni method is likely too conservative because it does not acknowledge any prior information. In our study we were informing our analysis using prior knowledge about variants that are known to be associated with steady‐state plasma lipid values to look for a differential effect in response to niacin. Therefore, the true power of the study is likely between these 2 threshold values. Second, we did not have access to another large cohort on chronic niacin treatment to replicate our findings, and our findings would require replication. It would be interesting to determine if our findings would replicate in the HPS2‐THRIVE study, which used a similar trial design.^8^ Third, we only evaluated the role of niacin on lipid‐dependent mechanisms on coronary disease risk, and we did not evaluate known lipid‐independent genes, as niacin has been shown to display anti‐inflammatory and antioxidant effects.^43^, ^44^, ^45^, ^46^ Last, there were a small number of black participants in the AIM‐HIGH study, so we were unable to examine genetic predictors of niacin response in this ethnic group. The lower participation is unfortunate in light of evidence that blacks are already known to have a significantly different response to niacin, at least in terms of triglyceride lowering^47^ and adverse effects.^48^

In conclusion, we have identified several genetic variants that are associated with the lipid response to niacin treatment. The association with genetic variation at *LIPC* encoding hepatic lipase is particularly interesting in that niacin has been previously suggested to modulate hepatic lipase activity. Although our results require replication, they represent the first pharmacogenetic study of lipid response to niacin and implicate *LIPC* and *CYP26A1* as potential mediators of niacin's effects on lipids and cardiovascular events.

## Sources of Funding

This work has been funded by the National Heart, Lung, and Blood Institute grant (R01HL086864) to D.J.R. S.T. was supported by a Clinical Research Program grant from the American Heart Association (11CRP7610016). AIM‐HIGH was supported by the National Heart, Lung, and Blood Institute (U01 HL081616 and U01 HL081649) and by an unrestricted grant from AbbVie, Inc. AbbVie had no role in the oversight or design of the study or in the analysis or interpretation of the data.

## Disclosures

None.

## Supporting information


**Table S1.** SNPs Tested and Association With Baseline Lipid Traits
