# Supplementary material for: Genetic Variants Associated With Plasma Lipids Are Associated With the Lipid Response to Niacin
Source: J Am Heart Assoc. 2018 Sep 29;7(19):e03488. doi: 10.1161/JAHA.117.008461 (PMC6404865; doi:10.1161/JAHA.117.008461)
Supplement: Supplementary file 1 — Table S1. SNPs Tested and Association With Baseline Lipid Traits [file JAH3-7-0e03488-s001.pdf]

SUPPLEMENTAL MATERIAL

Table S1. SNPs tested and association with baseline lipid traits.

SNP	Trait	Locus	Chr	MAF	N	Effect of minor allele Beta	SE	Stat	Pvalue	Proxy	GLGC SNP	r2
rs12748152	HDL	PIGV-NR0B2	1	0.08	2054	-0.006	0.053	-0.12	0.91			
rs12145743	HDL	HDGF-PMVK	1	0.34	2054	-0.018	0.030	-0.59	0.56			
rs1689800	HDL	ZNF648	1	0.37	2054	-0.015	0.030	-0.51	0.61			
rs4650994	HDL	ANGPTL1	1	0.47	2054	0.0004	0.028	0.01	0.99			
rs4660293	HDL	PABPC4	1	0.24	2054	-0.065	0.034	-1.89	0.06			
rs4846914	HDL	GALNT2	1	0.41	2054	-0.046	0.029	-1.58	0.11			
rs12328675	HDL	COBLL1	2	0.12	2054	-0.059	0.045	-1.30	0.19			
rs2972146	HDL	IRS1	2	0.34	2054	0.029	0.031	0.92	0.36			
rs13326165	HDL	STAB1	3	0.20	2054	-0.023	0.036	-0.63	0.53			
rs2013208	HDL	RBM5	3	0.48	2054	0.037	0.029	1.28	0.20			
rs2290547	HDL	SETD2	3	0.18	2054	0.055	0.038	1.45	0.15			
rs2606736	HDL	ATG7	3	0.38	2054	-0.011	0.030	-0.36	0.72			
rs6805251	HDL	GSK3B	3	0.38	2054	-0.049	0.030	-1.66	0.10			
rs10019888	HDL	C4orf52	4	0.17	2054	0.010	0.038	0.28	0.78			
rs13107325	HDL	SLC39A8	4	0.08	2054	-0.018	0.053	-0.35	0.73			
rs2602836	HDL	ADH5	4	0.44	2054	-0.030	0.029	-1.03	0.31			
rs3822072	HDL	FAM13A	4	0.48	2054	0.002	0.029	0.07	0.94			
rs6450176	HDL	ARL15	5	0.26	2054	-0.064	0.032	-1.98	0.05			
rs1936800	HDL	RSPO3	6	0.48	2054	0.028	0.029	0.98	0.33			
rs634869	HDL	CITED2	6	0.43	2054	-0.005	0.029	-0.18	0.85	Y	rs605066	1.00
rs17173637	HDL	TMEM176A	7	0.10	2054	-0.094	0.049	-1.93	0.05			
rs4142995	HDL	SNX13	7	0.39	2054	0.009	0.029	0.31	0.76			
rs4731702	HDL	KLF14	7	0.48	2054	0.001	0.028	0.02	0.99			
rs4917014	HDL	IKZF1	7	0.32	2054	0.005	0.031	0.16	0.87			
rs702485	HDL	DAGLB	7	0.45	2054	-0.005	0.029	-0.16	0.87			
rs2293889	HDL	TRPS1	8	0.44	2054	0.006	0.029	0.20	0.84			
rs9987289	HDL	PPP1R3B	8	0.09	2054	-0.028	0.050	-0.55	0.58			
rs1883025	HDL	ABCA1	9	0.26	2054	-0.012	0.032	-0.36	0.72			
rs581080	HDL	TTC39B	9	0.19	2054	-0.087	0.037	-2.36	0.02			
rs970548	HDL	MARCH8-ALOX5	10	0.25	2054	-0.017	0.033	-0.50	0.61			
rs11246602	HDL	OR4C46	11	0.12	2054	0.042	0.045	0.93	0.35			
rs12801636	HDL	KAT5	11	0.22	2054	-0.021	0.034	-0.61	0.54			
rs2923084	HDL	AMPD3	11	0.19	2054	-0.025	0.037	-0.66	0.51			
rs3136441	HDL	LRP4	11	0.12	2054	0.130	0.045	2.90	0.0037			
rs499974	HDL	MOGAT2-DGAT2	11	0.17	2054	-0.056	0.038	-1.47	0.14			
rs10850443	HDL	MVK	12	0.47	2054	-0.001	0.028	-0.05	0.96	Y	rs7134594	0.97
rs10773003	HDL	SBNO1	12	0.09	2043	0.048	0.051	0.94	0.35	Y	rs4759375	1.00
rs11057408	HDL	ZNF664	12	0.33	2054	0.009	0.030	0.30	0.77	Y	rs4765127	1.00
rs7134375	HDL	PDE3A	12	0.42	2054	0.049	0.029	1.67	0.10			
rs838880	HDL	SCARB1	12	0.31	2054	0.063	0.031	2.04	0.04			
rs4983559	HDL	ZBTB42-AKT1	14	0.39	2054	0.037	0.029	1.25	0.21			
rs1532085	HDL	LIPC	15	0.37	2054	0.106	0.030	3.58	0.00035			
rs2652834	HDL	LACTB	15	0.20	2054	-0.002	0.036	-0.06	0.95			
rs1121980	HDL	FTO	16	0.44	2054	-0.037	0.029	-1.26	0.21			
rs16942887	HDL	LCAT	16	0.12	2054	0.087	0.045	1.94	0.05			
rs2925979	HDL	CMIP	16	0.31	2054	0.001	0.031	0.04	0.97			
rs3764261	HDL	CETP	16	0.28	2054	0.178	0.032	5.60	2.40E-08			
rs11869286	HDL	STARD3	17	0.35	2054	0.013	0.030	0.42	0.68			
rs4129767	HDL	PGS1	17	0.48	2054	0.068	0.029	2.37	0.02			
rs4148008	HDL	ABCA8	17	0.32	2054	-0.055	0.031	-1.76	0.08			
rs12967135	HDL	MC4R	18	0.24	2054	-0.010	0.033	-0.30	0.76			
rs7239867	HDL	LIPG	18	0.17	2054	-0.077	0.038	-2.00	0.05			
rs17695224	HDL	HAS1	19	0.29	2054	0.038	0.032	1.20	0.23			
rs386000	HDL	LILRA3	19	0.20	2054	0.061	0.036	1.68	0.09			
rs7254882	HDL	ANGPTL4	19	0.48	2054	0.011	0.029	0.37	0.71			
rs737337	HDL	ANGPTL8	19	0.09	2054	-0.043	0.051	-0.85	0.40			
rs1800961	HDL	HNF4A	20	0.03	2054	-0.060	0.085	-0.71	0.48			
rs6065906	HDL	PLTP	20	0.19	2054	0.027	0.036	0.73	0.47			
rs181362	HDL	UBE2L3	22	0.20	2054	-0.036	0.036	-1.02	0.31			
rs2479409	LDL	PCSK9	1	0.35	2054	-0.006	0.032	-0.18	0.85			
rs267733	LDL	ANXA9-CERS2	1	0.15	2054	-0.052	0.046	-1.13	0.26			
rs629301	LDL	SORT1	1	0.20	2054	-0.153	0.039	-3.95	8.05E-05			
rs10490626	LDL	INSIG2	2	0.07	2054	-0.141	0.060	-2.36	0.02			
rs1250229	LDL	FN1	2	0.27	2054	0.006	0.035	0.18	0.85			
rs1367117	LDL	APOB	2	0.30	2054	0.035	0.034	1.02	0.31			
rs2030746	LDL	LOC84931	2	0.42	2054	0.033	0.032	1.03	0.30			
rs2710642	LDL	EHBP1	2	0.32	2054	-0.023	0.033	-0.70	0.48			
rs4299376	LDL	ABCG5/8	2	0.31	2054	0.082	0.034	2.44	0.01			
rs17404153	LDL	ACAD11	3	0.12	2054	-0.017	0.048	-0.35	0.73			
rs7640978	LDL	CMTM6	3	0.08	2054	0.011	0.056	0.20	0.84			
rs4530754	LDL	CSNK1G3	5	0.44	2054	-0.020	0.032	-0.64	0.52			
rs1564348	LDL	LPA	6	0.16	2054	-0.025	0.043	-0.59	0.55			
rs1800562	LDL	HFE	6	0.06	2054	0.024	0.063	0.38	0.70			
rs3757354	LDL	MYLIP	6	0.21	2054	-0.055	0.038	-1.44	0.15			
rs4722551	LDL	MIR148A	7	0.17	2054	-0.010	0.041	-0.23	0.81			
rs10102164	LDL	SOX17	8	0.20	2054	-0.001	0.039	-0.02	0.98			
rs7832643	LDL	PLEC1	8	0.41	2054	0.008	0.032	0.25	0.80	Y	rs11136341	0.81
rs9411489	LDL	ABO	9	0.20	2054	0.058	0.038	1.53	0.13			
rs11220462	LDL	ST3GAL4	11	0.13	2054	0.019	0.046	0.42	0.68			
rs4942486	LDL	BRCA2	13	0.48	2054	0.039	0.031	1.25	0.21			
rs8017377	LDL	NYNRIN	14	0.47	2054	-0.056	0.031	-1.78	0.08			

rs1801689	LDL	APOH-PRXCA	17	0.03	2054	0.040	0.090	0.45	0.66			
rs6504872	LDL	OSBPL7	17	0.49	2054	0.001	0.031	0.02	0.99			
rs4420638	LDL	APOE	19	0.18	2054	0.022	0.041	0.54	0.59			
rs6511720	LDL	LDLR	19	0.11	2054	-0.087	0.051	-1.72	0.09			
rs2223745	LDL	TOP1	20	0.48	2054	0.050	0.032	1.58	0.11			
rs2328223	LDL	SNX5	20	0.19	2054	-0.071	0.040	-1.79	0.07			
rs364585	LDL	SPTLC3	20	0.39	2054	-0.063	0.032	-2.00	0.05			
rs5763662	LDL	MTMR3	22	0.02	2054	0.065	0.114	0.57	0.57			
rs1077514	Total Chol	ASAP3	1	0.14	2054	0.031	0.044	0.71	0.48			
rs1556562	Total Chol	EVI5	1	0.21	2054	-0.001	0.038	-0.02	0.99	Y	rs7515577	1.00
rs2642442	Total Chol	MOSC1	1	0.31	2054	-0.071	0.033	-2.15	0.03			
rs558971	Total Chol	IRF2BP2	1	0.47	2054	-0.039	0.031	-1.29	0.20	Y	rs514230	0.96
rs11563251	Total Chol	UGT1A1	2	0.11	2054	0.027	0.050	0.54	0.59			
rs11694172	Total Chol	FAM117B	2	0.24	2054	0.006	0.036	0.18	0.86			
rs2287623	Total Chol	ABCB11	2	0.40	2054	0.010	0.031	0.31	0.76			
rs7570971	Total Chol	RAB3GAP1	2	0.37	2054	-0.020	0.030	-0.65	0.51			
rs11709504	Total Chol	RAF1	3	0.19	2054	0.062	0.041	1.52	0.13	Y	rs2290159	0.81
rs13315871	Total Chol	PXK	3	0.09	2054	0.038	0.054	0.71	0.47			
rs12916	Total Chol	HMGCR	5	0.41	2054	0.045	0.031	1.46	0.14			
rs6882076	Total Chol	TIMD4	5	0.36	2054	0.005	0.032	0.14	0.89			
rs2758886	Total Chol	KCNK17	6	0.30	2054	0.034	0.033	1.02	0.31			
rs2814982	Total Chol	C6orf106	6	0.11	2054	-0.017	0.049	-0.35	0.73			
rs3177928	Total Chol	HLA	6	0.15	2054	-0.038	0.043	-0.88	0.38			
rs9376090	Total Chol	HBS1L	6	0.25	2054	0.012	0.034	0.36	0.72			
rs12670798	Total Chol	DNAH11	7	0.24	2054	0.053	0.036	1.48	0.14			
rs1997243	Total Chol	GPR146	7	0.16	2054	0.050	0.042	1.19	0.23			
rs2072183	Total Chol	NPC1L1	7	0.23	2054	0.068	0.037	1.86	0.06			
rs4738684	Total Chol	CYP7A1	8	0.34	2054	-0.010	0.033	-0.30	0.77	Y	rs2081687	0.91
rs3780181	Total Chol	VLDLR	9	0.07	2054	-0.069	0.061	-1.13	0.26			
rs10904908	Total Chol	VIM-CUBN	10	0.43	2054	0.051	0.031	1.63	0.10			
rs2255141	Total Chol	GPAM	10	0.31	2054	-0.032	0.034	-0.94	0.35			
rs10128711	Total Chol	SPTY2D1	11	0.27	2054	-0.030	0.034	-0.87	0.38			
rs11603023	Total Chol	PHLDB1	11	0.44	2054	-0.025	0.030	-0.81	0.42			
rs7941030	Total Chol	UBASH3B	11	0.39	2054	-0.002	0.031	-0.06	0.95			
rs11065987	Total Chol	BRAP	12	0.45	2054	-0.082	0.031	-2.62	0.0090			
rs1169288	Total Chol	HNF1A	12	0.31	2054	0.090	0.033	2.69	0.0072			
rs4883201	Total Chol	PHC1-A2ML1	12	0.11	2054	-0.015	0.049	-0.31	0.75			
rs2000999	Total Chol	HPR	16	0.22	2054	0.030	0.037	0.81	0.42			
rs314253	Total Chol	DLG4	17	0.34	2054	-0.044	0.033	-1.32	0.19			
rs10401969	Total Chol	CILP2	19	0.06	2054	0.007	0.064	0.10	0.92			
rs492602	Total Chol	FLJ36070	19	0.49	2054	0.007	0.031	0.22	0.82			
rs2277862	Total Chol	ERGIC3	20	0.15	2054	0.013	0.043	0.30	0.76			
rs2902940	Total Chol	MAFB	20	0.30	2054	-0.031	0.034	-0.91	0.37			
rs138777	Total Chol	TOM1	22	0.34	2054	0.021	0.032	0.66	0.51			
rs4253772	Total Chol	PPARA	22	0.11	2054	0.138	0.050	2.76	0.0059			
rs2131925	TG	ANGPTL3	1	0.33	2054	-0.089	0.033	-2.68	0.0074			
rs1260326	TG	GCKR	2	0.43	2054	0.112	0.031	3.59	0.0003			
rs645040	TG	MSL2L1	3	0.22	2054	-0.028	0.038	-0.74	0.46			
rs442177	TG	KLHL8	4	0.40	2054	-0.050	0.031	-1.59	0.11			
rs6831256	TG	LRPAP1	4	0.44	2054	0.053	0.031	1.70	0.09			
rs9686661	TG	MAP3K1	5	0.21	2054	0.021	0.038	0.54	0.59			
rs719726	TG	RSPO3	6	0.43	2054	-0.005	0.031	-0.16	0.87			
rs998584	TG	VEGFA	6	0.50	2054	-0.021	0.031	-0.70	0.49			
rs13238203	TG	TYW1B	7	0.03	2054	-0.008	0.093	-0.08	0.93			
rs17145738	TG	MLXIPL	7	0.11	2054	-0.037	0.050	-0.74	0.46			
rs38855	TG	MET	7	0.48	2054	0.033	0.031	1.05	0.30			
rs11776767	TG	PINX1	8	0.38	2054	0.032	0.032	0.99	0.32			
rs12678919	TG	LPL	8	0.07	2054	-0.131	0.060	-2.20	0.03			
rs2954029	TG	TRIB1	8	0.44	2054	-0.113	0.031	-3.61	0.00032			
rs10761741	TG	JMJD1C	10	0.44	2054	0.045	0.031	1.44	0.15	Y	rs10761731	0.99
rs1832007	TG	AKR1C4	10	0.15	2054	-0.081	0.044	-1.83	0.07			
rs2068888	TG	CYP26A1	10	0.44	2054	-0.030	0.031	-0.96	0.34			
rs174546	TG	FADS1-2-3	11	0.34	2054	0.072	0.033	2.19	0.03			
rs11613352	TG	LRP1	12	0.22	2054	0.000	0.038	-0.01	0.99			
rs2412710	TG	CAPN3	15	0.02	2054	-0.012	0.104	-0.12	0.91			
rs2929275	TG	FRMD5	15	0.05	2054	0.056	0.067	0.84	0.40	Y	rs2929282	0.97
rs3198697	TG	PDXDC1	16	0.39	2054	-0.043	0.032	-1.36	0.17			
rs9930333	TG	FTO	16	0.44	2054	0.077	0.031	2.46	0.01			
rs8077889	TG	MPP3	17	0.22	2054	0.067	0.038	1.75	0.08			
rs7248104	TG	INSR	19	0.41	2054	-0.016	0.032	-0.51	0.61			
rs731839	TG	PEPD	19	0.34	2054	0.046	0.033	1.39	0.16			
rs5756931	TG	PLA2G6	22	0.39	2054	-0.026	0.031	-0.84	0.40			

rs394352	Lp(a)	SLC22A3	6	0.29	2054	-0.245	0.034	-7.22	7.30E-13
rs2504927	Lp(a)	SLC22A3	6	0.43	2054	-0.250	0.032	-7.90	4.59E-15
rs4252109	Lp(a)	PLG	6	0.29	2054	-0.290	0.034	-8.62	1.32E-17
rs539298	Lp(a)	SLC22A3	6	0.47	2054	-0.273	0.031	-8.84	1.99E-18
rs7769879	Lp(a)	SLC22A3	6	0.39	2054	0.351	0.032	11.11	7.03E-28
rs986666	Lp(a)	SLC22A3	6	0.20	2054	-0.155	0.039	-3.95	8.10E-05
rs2457561	Lp(a)	SLC22A3	6	0.19	2054	-0.154	0.041	-3.81	0.00015
rs3798221	Lp(a)	LPA	6	0.19	2054	-0.282	0.039	-7.18	1.00E-12

GLGC= Global Lipids Genetic Consortium
